# Systemic Lupus Erythematosus Presenting as Pulmonary Embolism After Liposuction: A Clinical Conundrum

**DOI:** 10.7759/cureus.16076

**Published:** 2021-06-30

**Authors:** Aneesh Kumar, Haider Ghazanfar, Faryal Altaf

**Affiliations:** 1 Internal Medicine, BronxCare Hospital Center, New York, USA; 2 Internal Medicine, BronxCare Health System, New York, USA; 3 Internal Medicine, Continental Medical College, Lahore, PAK

**Keywords:** pulmonary embolism, anticoagulation, systemic lupus erythematosus, liposuction, galectin-3-binding protein, fat embolism, latent lupus

## Abstract

Systemic lupus erythematosus (SLE) is an autoimmune disorder with a wide array of presentations and a predilection to affect women of certain ethnic backgrounds. The hallmark of the disease is multisystem involvement, dispersed in time and severity. Usual pulmonary involvement includes pleuritis, pleural effusions, pneumonitis, shrinking lung syndrome, pulmonary hypertension, and alveolar hemorrhage. Pulmonary embolism (PE) is a relatively unusual presentation of SLE. We present the case of a 28-year-old Hispanic female who presented with shortness of breath and chest pain after liposuction and was found to have a PE. Fat embolism was ruled out. The absence of overt signs and symptoms and traditional risk factors prompted a fragmentary workup. This led to the detection of antibodies sensitive and specific for SLE, in the absence of overt signs and symptoms. We revive the concept of latent lupus, a condition construed as early lupus. Since our patient was lost to follow up, we were unable to complete workup for SLE, but firmly suspect direct causation between SLE and PE. Further studies are needed to establish pathogenesis in order to facilitate early diagnosis and prevent morbidity and mortality from PE.

## Introduction

Pulmonary embolism (PE) is the third most common cause of cardiovascular mortality. Traditional risk factors include immobilization, active malignancy, trauma, congestive heart failure, and others, which account for 74% of cases of venous thromboembolism (VTE), leaving 25% of cases unexplained [1]. Systemic lupus erythematosus (SLE) is an autoimmune disorder with a variety of manifestations. SLE usually presents with fatigue, arthralgia, arthritis, myalgia, and weight loss, but outside the musculoskeletal system, the pulmonary system is the next most commonly affected [2]. SLE and other autoimmune diseases have been associated with increased risk of VTE, and this report shows a likely association between SLE and PE.

## Case presentation

A 28-year-old Hispanic female with no comorbidities presented with sudden-onset, sharp chest pain, and shortness of breath for one day. She denied any cough, fever, chills, hemoptysis, calf swelling, or leg pain. She reported that she underwent liposuction at a local clinic two weeks prior to the onset of symptoms. She reported no family history of bleeding, clotting, or rheumatologic disorders, no drug allergies, and toxic habits. She had one healthy baby delivered vaginally five years ago. She was never on oral contraceptive medications.

**Figure 1:**
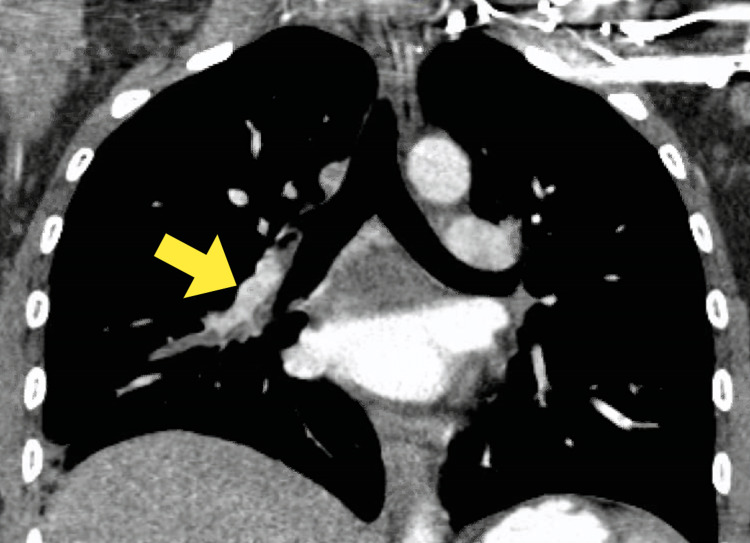
CT scan of the chest showing pulmonary embolism within the segmental and subsegmental branches of the right anterior lung basal arteries

On presentation, her pulse rate was 116 beats per minute and oxygen saturation was 98% on room air. Her body mass index (BMI) was 29. On examination, her breath sounds were decreased bilaterally due to pain. Her right lower extremity was swollen compared to the left lower extremity. Her neurological examination was normal and no skin rashes were noted. Her electrocardiogram revealed Q waves in lead III and S wave in lead I, in the setting of sinus tachycardia. Her laboratory tests showed a prothrombin time of 11.2 seconds, activated partial thromboplastin time of 51.9 seconds, D-dimer of 424 ng/mL, troponin T <12 ng/L, and pro-brain natriuretic peptide of 91 pg/mL. The Wells score was calculated to be 6. Doppler venous ultrasonography did not reveal any deep venous thrombus. Computerized tomography (CT) imaging of the chest revealed partially occlusive pulmonary emboli within the segmental and subsegmental branches of the right anterior lung basal arteries. This has been shown in Figure 1.

**Figure 2:**
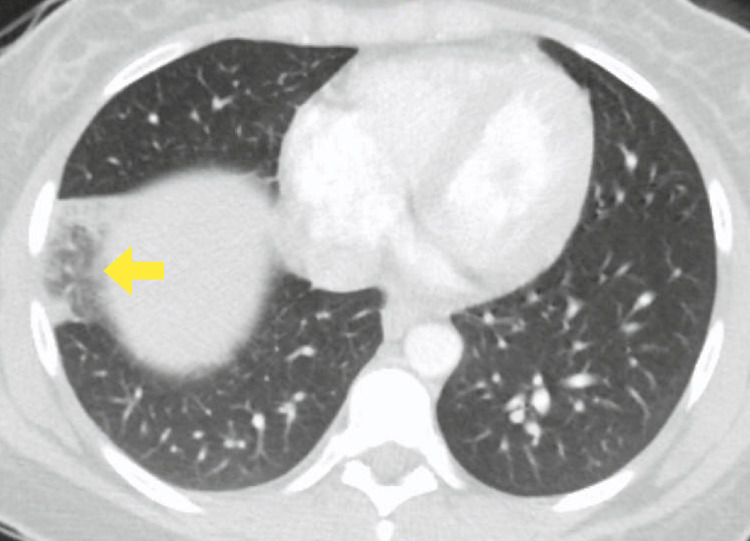
Focal airspace opacities within the segment, suggestive of a pulmonary infarct

The CT scan also showed focal airspace opacities within the anterior basal segment of the right lower lobe, suggestive of a pulmonary infarct. This has been shown in Figure 2.

She was started on a heparin drip, which was later bridged to apixaban. She improved symptomatically and her heart rate normalized. Hypercoagulable workup has been presented in Table 1.

**Table 1:** Hypercoagulable workup

Test parameter	Result	Reference
Antinuclear antibody screen	Positive (1:1280)	Negative
Antibody to double-stranded deoxyribonucleic acid	10 IU/mL	< or= 4 IU/mL
Serum total homocysteine	9.8 umol/l	<10.4umol/l
Protein C functional assay	141%	70-180%
Protein S functional assay	79%	60-140%
Antithrombin III assay	102%	80-135%
Prothrombin gene G20210A mutation	Not detected	Not detected
Dilute Russell viper venom time screen	Negative	Negative
Cardiolipin antibody screen	Negative	Negative
Beta 2 glycoprotein screen	Negative	Negative
Phosphatidylserine antibody	Negative	Negative

She was discharged on a three-month supply of apixaban and was advised to follow up in the hematology and rheumatology clinics for repeat testing as per current guidelines. However, the patient was lost to follow-up.

## Discussion

The annual incidence of PE in the United States has been estimated to be around 150,000 to 250,000 cases, with a death rate of 60,000 to 100,000 annually [3]. The pathogenesis is related to Virchow’s triad. The presenting symptoms include dyspnea (73%), pleuritic chest pain (47%), cough (43%), calf or thigh pain (up to 42%), calf or thigh swelling (up to 39%), and wheezing (31%) [4].

Patients with SLE have a 2.2 to 3.3 times higher relative risk of death than the general population [5]. The pathogenesis of the disease is still under investigation but revolves around abnormalities in innate and acquired immunity [6]. Due to heterogeneity, accurate diagnosis of SLE has always been a clinical challenge. The latest criteria for SLE comes from the European League Against Rheumatism (EULAR)/American College of Rheumatology classification, which has been reported to have a sensitivity of 96.1% and specificity of 93.4% [7].

Our patient had none of the usual signs and symptoms of SLE [2]. Liposuction has been reported as a risk factor for fat embolism [8]. The observed CT findings in fat embolism include opacities, nodules, and areas of consolidation, mostly in the upper lobes [9]. Although the clinical diagnosis of fat embolism was suspected in our patient, CT imaging suggestive of thrombus ruled out fat embolism. Furthermore, the absence of neurological changes (59% prevalence) and rash (33% prevalence) helped exclude this diagnosis [10]. An autoimmune workup was requested out of academic inquisitiveness, which was positive for high titer ANA and anti-dsDNA antibodies. Our patient did not have a complete workup for SLE, including complement levels and estimation of proteinuria. Hence, the EULAR criteria for SLE diagnosis were not applicable to our patient.

We want to revisit the concept of “latent lupus” at this juncture, which is believed to be an evolutionary phase of SLE, with a high risk of development of SLE in a few years [11]. Routine monitoring of patients who do not meet full criteria currently can aid in early diagnosis and management [12]. The overall risk of pulmonary embolism in the first year after admission for an autoimmune disorder is reported to be 6.38 (95% CI 6.19-6.57). The risk was particularly high with SLE at 10.23 (95% CI 8.31-12.45). There is a suggestion that these autoimmune conditions should be managed as hypercoagulable states. Also, the risk of VTE seems to reduce over the years since diagnosis, further emphasizing the importance of a timely diagnosis [13]. There are currently no established predictors of VTE in SLE, except for a pilot study that followed levels of galectin-3-binding protein (G3BP). G3BP is believed to induce a prothrombotic state by activating type I interferon. The reported hazard ratio for increased risk of VTE was 1.18 (95% CI: 1.05-1.33, p=0.007) [14].

## Conclusions

Through this report, we aim to emphasize that acute pulmonary embolism could be the initial presentation of systemic lupus erythematosus and other autoimmune disorders. Physicians should keep a low threshold to suspect SLE and other autoimmune disorders, even in the absence of overt clinical features, in patients with unexplained VTE. Further studies are needed to establish the pathogenesis and to prevent thromboembolic events in patients with autoimmune disorders.
